# Generation of Customizable Micro-wavy Pattern through Grayscale Direct Image Lithography

**DOI:** 10.1038/srep21621

**Published:** 2016-02-23

**Authors:** Ran He, Shunqiang Wang, Geoffrey Andrews, Wentao Shi, Yaling Liu

**Affiliations:** 1 Department of Mechanical Engineering and Mechanics, Lehigh University, Bethlehem, PA 18015, USA; 2 Bioengineering Program, Lehigh University, Bethlehem, PA 18015, USA

## Abstract

With the increasing amount of research work in surface studies, a more effective method of producing patterned microstructures is highly desired due to the geometric limitations and complex fabricating process of current techniques. This paper presents an efficient and cost-effective method to generate customizable micro-wavy pattern using direct image lithography. This method utilizes a grayscale Gaussian distribution effect to model inaccuracies inherent in the polymerization process, which are normally regarded as trivial matters or errors. The measured surface profiles and the mathematical prediction show a good agreement, demonstrating the ability of this method to generate wavy patterns with precisely controlled features. An accurate pattern can be generated with customizable parameters (wavelength, amplitude, wave shape, pattern profile, and overall dimension). This mask-free photolithography approach provides a rapid fabrication method that is capable of generating complex and non-uniform 3D wavy patterns with the wavelength ranging from 12 μm to 2100 μm and an amplitude-to-wavelength ratio as large as 300%. Microfluidic devices with pure wavy and wavy-herringbone patterns suitable for capture of circulating tumor cells are made as a demonstrative application. A completely customized microfluidic device with wavy patterns can be created within a few hours without access to clean room or commercial photolithography equipment.

The development of practical and cost-effective methods of producing patterned microstructures is currently of great interest, especially in the field of surface studies^1^, cell adhesion^2^, and microfluidic^3^. The wavy pattern is highly desirable features, as it is eminently useful for biological applications^4^,^5^ and for adjusting surface properties such as adhesion^6^,^7^, friction^6^, and hydrophobicity^8^. A standard technique to create micro-wavy features is depositing thin metal films onto polydimethylsiloxane (PDMS) substrates with some degree of thermally-induced pre-strain^9^. Releasing of the strain is capable of generating wavy patterns with a uniform wavelength. Success has also been found in pre-straining substrates using mechanical force. Studies by Yang’s group found that a sequential mechanical stretching and unstretching of an oxidized PDMS membrane was sufficient to induce a highly ordered, uniform herringbone pattern^8^.

However, methods which rely on pre-straining a substrate are inherently limited in the wave shapes, dimensions, and diversity of the pattern profiles. In particular, the sequential and unequal biaxial stretching method cannot produce features with a wavelength greater than 50 μm, which creates a clear barrier for making large-scale wavy patterns^8^. In addition, the ratio of amplitude to wavelength reaches a limit at approximately 30%, greatly restricting the wave shapes which can be created. Furthermore, there is a more obvious limitation in that only select profiles, namely wavy and wavy-herringbone patterns, can be produced by the pre-strain method. The standard method of applying thin metal films is also incapable of producing a pattern with a wavelength beyond the range of 20–50 μm^9^. This metal deposition method also has significant shortcomings in cost and complexity, as it typically uses electron beam evaporation to deposit 50-nm-thick layers of gold with a 5-nm adhesion interlayer of titanium or chromium, requiring a complex fabrication process and high material cost. There are a few other methods of creating micro-wavy patterns based on the lithography approach. Crosby’s group has developed a method of generating wrinkled patterns in UV-cured polymer films, using a diffusion induced oxygen concentration gradient to inhibit polymerization during UV-curing and form an uncured liquid layer that spontaneously swells the film^10^. This approach has generated wrinkle patterns with controllable wavelength and amplitude, distribution of the wavy structures is random. Thus, the method is incapable of creating a micro-wavy pattern with specific profiles, such as wavy-herringbone patterns.

As an alternative, the grayscale lithography method allows for the rapid fabrication of three-dimensional microstructures with greatly reduced complexity^11^,^12^,^13^. Whitesides’ group used a grayscale mask in photolithography to create microlens^14^. However, generating the photomask is time consuming and not cost-effective, making it difficult and impractical to customize the patterns for a specific application.

Another method relies upon the use of digital micro-mirror devices (DMD), which are capable of adjusting the hue of each pixel in an image; this is known as Digital Light Processing (DLP). Using a standard grayscale color mapping, 256 different light levels are thus possible, creating a highly capable curing device when the DMD chip is combined with an appropriate light source^15^. As such, an image with multiple grayscale levels can be used to directly create three-dimensional features in a single exposure. Park’s group achieved success in fabricating three-dimensional structures using this type of mask-free lithography method^16^. Kwon’s group utilized a similar approach as an *in situ* polymerization technique to generate gradational micropatterning^17^. His group also developed a method that utilizes the light overlap to fabricate microstructures as polymer microtaggants for anti-counterfeiting of drugs^18^. However, to generate precisely customizable microstructures, both the light distortion effects and mathematical models need to be considered comprehensively to predict intended results.

This paper outlines a direct image lithography technique which uses grayscale mapping in conjunction with a mathematical model for light distortion to create a wavy pattern with diverse geometries in a precise and controllable manner.

## Results and Discussion

### UV Induced Polymerization Model

In UV radiation curing processes, light absorbed by the photoinitiator generates free radicals, which induce further polymerization and cross-linking. The three basic steps of chain-growth polymerization are initiation, propagation, and termination. According to the Jacobs model^19^, a photocurable resin is cured only if the exposure received by each point is greater than the threshold exposure of polymerization. On the other hand, Beer Lambert’s law of absorption provides a relation between curing depth, exposure energy and depth of penetration of the resin^20^.

**Figure 1:**
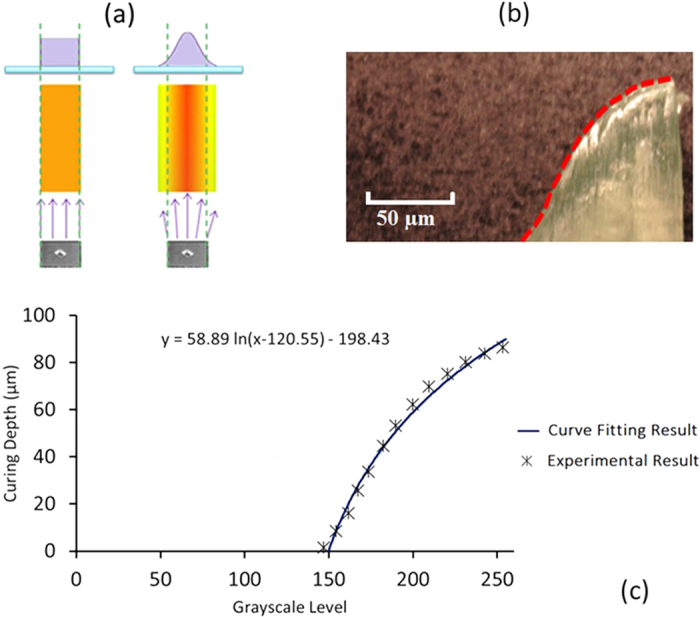
Analysis of the resin polymerization process. (**a**) Theoretical curing model and actual curing result of the photocurable resin under the UV exposure of one pixel area. (**b**) Profile of photocurable resin cured under the exposure of grayscale gradient. (**c**) Curve fitting of the experimental result.

In this paper, a direct image lithography system is developed to project a target pattern onto the polymer resin. The key element of this system is the DMD, which creates the pattern by rotating an array of micro-mirrors to reflect the UV light and harden the photocurable resin. The pixels projected on the resin which correspond to those individual mirrors determine the theoretical resolution of the pattern. In reality, the energy of a single pixel projected on the surface of resin is distributed uniformly in the desired area. In the real situation, the energy distribution of each pixel follows a Gaussian distribution and diffuses to the nearby area due to optical dispersion, spherical aberration, astigmatism, spatial incoherence and distortion^15^. Figure 1(a) illustrates the difference between the theoretical curing assumption and the actual curing result.

This Gaussian distribution effect can be utilized together with the grayscale effect to generate three dimensional patterns with smooth surfaces. A digital grayscale image has a certain value for each pixel that carries the light intensity information, varying from black at the weakest intensity (with grayscale value = 0) to white at the strongest (with grayscale value = 255). The micro-mirror on the DMD chip is capable of displaying 256 grayscale levels by adjusting the frequency with which each mirror switches between “ON” and “OFF”. When projecting UV light to cure the photocurable resin, a higher grayscale value (corresponding to greater light intensity) will lead to a greater curing depth. Figure 1(b) shows a side view of the polymerized photocurable resin under the exposure of grayscale gradient from 0 to 255. Figure 1(c) shows a curve fitted to the experimental result, providing an equation that can express the relationship between grayscale level G and curing depth D as^20^:


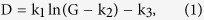


where k_1_, k_2_, and k_3_ are 58.89, 120.55 and 198.43, respectively. The values of k_1_, k_2_, and k_3_ are dependent on the type of photocurable material and the exposure time. This equation provides a method of controlling the curing thickness by choosing proper grayscale values. Based on the optical system and photocurable material used in this paper, the curing threshold grayscale value is 150. An image with grayscale level less than 150 will not polymerize the liquid resin regardless of how long the exposure time is.

**Figure 2:**
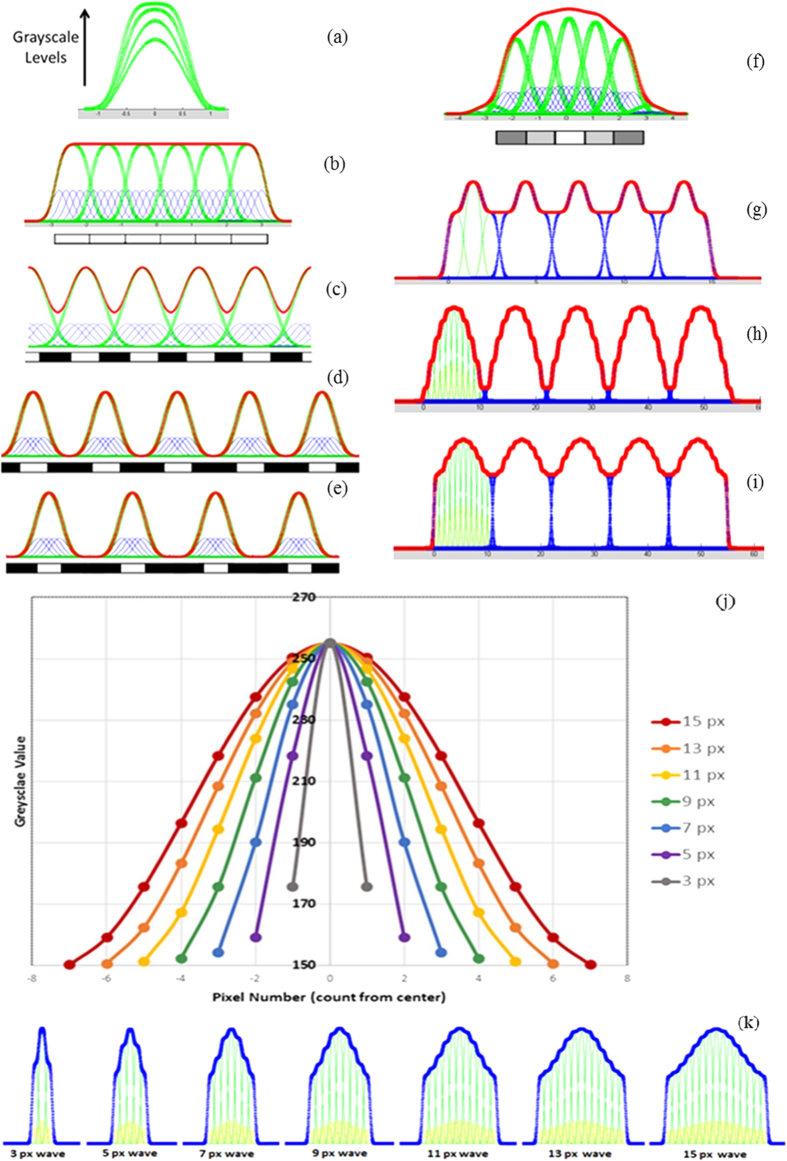
Mathematical model of curing shapes. (**a**) Variation of single pixel curing models at increased grayscale levels. (**b**) The curing model under the exposure of a pattern with continuous pixels at the same grayscale values of 200. (**c**–**e**) The curing model under the exposure of a pattern with gray pixels (with grayscale value of 200) alternating with black pixels (with grayscale value of 0). The numbers of the black pixels between two nearby gray pixels are 1 in (**c**), 2 in (**d**) and 3 in (**e**,**f**) The curing model of a single wave containing five grayscale pixels. (**g**–**i**) Curing models of wave series containing multiple grayscale pixels in each wave. The numbers of grayscale pixels in each wave are 3 in (**g**), 9 in (**h**) and 11 in (**i**), respectively. (**j**) Single wave curing models according to different pixel numbers in each wave. (**k**) Parameters of grayscale values and number of pixels applied in (**j**). (In (**a**–**i**), Green curves represent the curing shape of a single pixel; Red curves represent the final curing shapes of the entire exposure pattern. In (**j**), blue curves represent curing shapes of each whole wave).

In the mathematical model, a row of DMD micro-mirrors has been selected to study the curing shape generated under different grayscale levels. Side view of a single pixel’s curing shape with increasing of grayscale level is illustrated in Fig. 2(a). As the grayscale level increases, the curing depth increases and the top part of the cured shape gets more flat.

A curing model of a single white pixel with grayscale value of 255 can be expressed as:


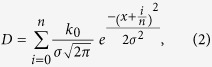


where D is the curing depth *x* is the horizontal coordinate position, and *k*_0_ is a factor of proportionality related with the material system and exposure time. In the current system setup, the values of k_0_, σ and n are 2.87, 0.15 and 10, respectively.

When a pattern containing multiple pixels is projected, the actual light intensity of a certain projected point is the summation of the light intensities contributed by the pixel projecting directly onto that point as well as by nearby pixels. As shown in Fig. 2(b), when an image of several continuous pixels in a row with a grayscale value of 200 is projected, a planar top surface with smooth edges is formed. The diffusion of light among neighboring pixels compensates for the lower energy near the edge of each pixel; the result is that the gaps in the cumulative light profile are filled in, forming a flat surface. A wave form can be generated by projecting a pattern of gray pixels (with grayscale value from 150 to 255) alternating with black pixels (with grayscale value 0). The gray pixel can be called “effective” pixels while the black pixels are called “ineffective” pixels. Figure 2(c–e) show the curing shapes formed with different numbers of “ineffective” pixels between two effective pixels. An approximately sinusoidal wavy pattern is generated when there is only one “ineffective” pixel between two “effective” pixels. By increasing the number of sandwiched “ineffective” pixels, the bottom part of the wave becomes wider and more flat until a section with a completely flat bottom is formed. Once two nearby “effective” pixels are separated by a certain distance, their energy can no longer affect the entire area between them. As a result, the planar bottom is formed - this limits the wavelengths for which a smoothly curved wavy pattern is possible for pixels at a single grayscale level. To create a wavy pattern with unlimited wavelength and shape, the curing system must be able to create a wave using multiple pixels at different grayscale levels. This paper develops a method of implementing multiple simultaneous grayscale levels to fabricate micro-wavy patterns across a wide range of wavelengths.

With multiple grayscale levels, the wavelength depends primarily on the number of grayscale pixels for each wave rather than size or influential are of each pixel. In Fig. 2(f), a single wave is formed by five adjacent grayscale pixels. In Fig. 2(g–i), three series of waves with various wavelengths and shapes are formed by introducing multiple grayscale pixels and values. Figure 2(j) shows a simulation of seven groups of wave shapes with different pixel numbers and grayscale values that decrease from the peak to the bottom according to the grayscale parameters given in Fig. 2(k). Each point represents a pixel with a specific grayscale value. In this model, the wavelength is determined by the number of pixels, while the amplitude is determined by the difference between the maximum and minimum grayscale value in each wave. The shape of the wave (triangular, sinusoid, square, etc.) can be specified by using a particular combination of grayscale values.

### Programmable Pattern Generation

**Figure 3:**
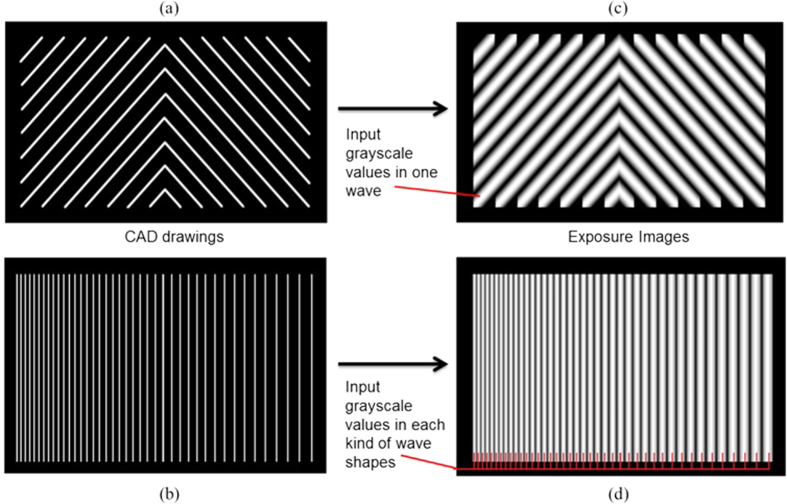
Pattern generating process. (**a**) CAD drawings of uniformly distributed herringbone lines. (**b**) CAD drawings of a column of lines with the gradient determined by the gap distance. (**c,d**) The grayscale exposure image generated from drawing (**a,b**), respectively.

The grayscale image necessary to produce a given profile is generated via a specialized pattern generation program. As shown in Fig. 3(a,b), a pattern of 2D lines is drawn using CAD software and saved as a .dxf format file. The distance between two lines determines the wavelength of the micro-features - the pattern generation code reads the .dxf file and creates a grayscale exposure image which is used in the lithography process to form the desired features. The exposure image can be created by inputting different combinations of grayscale values for different lines in the CAD file. Once the target wavelength and wave shape are decided, the mathematical model can provide grayscale values to be input into the pattern generation code. The output image, such as Fig. 3(c,d) can be directly used for exposure through the projecting system. This pattern generation program is capable of generating complex grayscale images for a variety of desired shapes.

## Material

The photocurable material created in this paper is a mixture of Etermer 6210G (a modified epoxy acrylate, Eterner Chem. Co.), 1,6-hexanediol diacrylate (HDDA, BASF Co.), Tripropylene Glycol Diacrylate (TPGDA, BASF Co.), ethoxylatedtrimethylolpropanetriacrylate (TMP3EOTA, Eterner Chem. Co.) and lauryl acrylate (LA, Esstech Co.), using the photoinitiators, Irgacure 754 (a mixture of Oxy-phenyl-acetic acid 2-[2-oxo-2-phenyl-acetoxy-ethoxy]-ethyl ester and Oxy-phenyl-acetic acid 2-[2-hydroxy-ethoxy]-ethyl ester) and Irgacure 4265 (a mixture of Diphenyl (2,4,6-trimethylbenzoyl-phosphine oxide and 2-Hydroxy-2-methyl-1-phenyl-propan-1-one)) from BASF. Tinopal OB (BASF) is utilized as a UV absorber.

## System Setup

**Figure 4:**
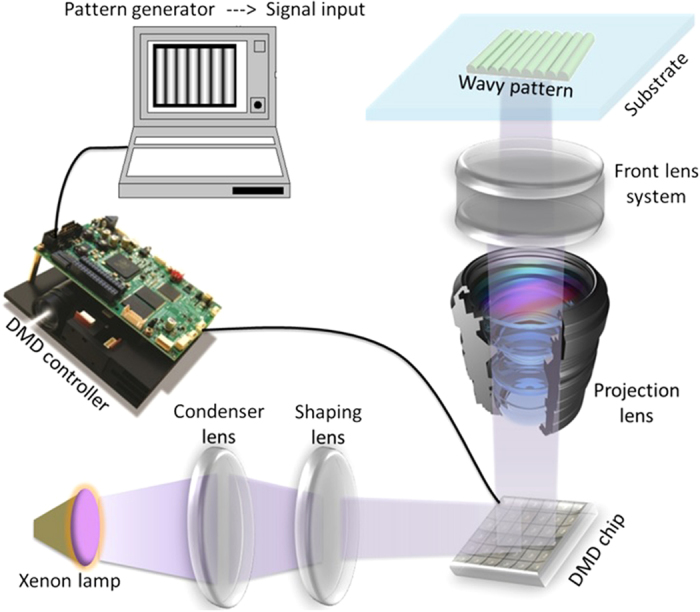
The schematic diagram of the wavy pattern projection system.

The micro-pattern generation system has an optical platform including a light source, condensing lens, shaping lens, a DMD chip (digital micro-mirror device, SXGA+, Texas Instruments, USA), projection lens, and front lens as shown in Fig. 4. The optical projection system is designed for projecting grayscale images, and the SXGA+ DMD chip is capable of instantaneous exposure (with a minimum exposure time of 10^−4^ second). The front lens system is installed to fix the image distortion as well as adjust the printing resolution. A xenon lamp is used as the light source with a main wavelength ranges from 330 nm to 580 nm. However, only the UV portion of the projection spectrum (ranging from 330 nm to 390 nm wavelength) is effective in the polymerization process, because the reaction range of the photocurable resin is less than 390 nm. The optical system can reduce size of pixels projected on the glass substrate to a minimum size of 10 μm, which determines the minimum wavelength of the wavy-pattern that can be fabricated with the apparatus.

## Results and Analysis

**Figure 5:**
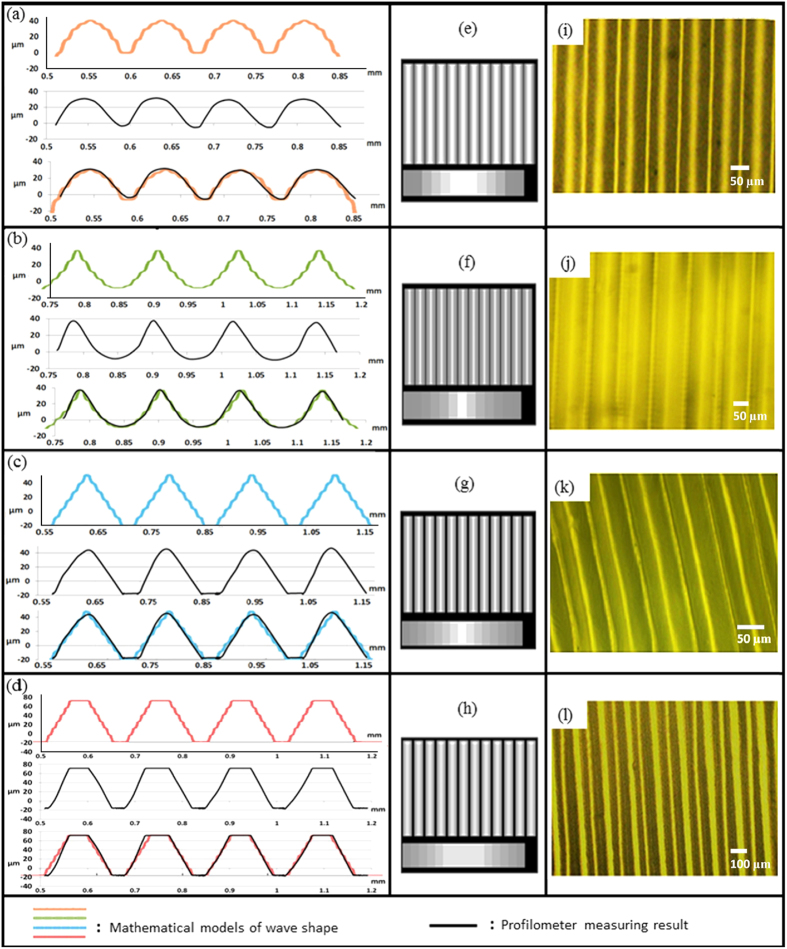
Comparison between experimental result and mathematical model. (**a**–**d**) Mathematical curing models, profilometer measurement results, and the comparison between the exposure images in grayscale (**e**–**h**) and the digital microscope images of fabricated wavy patterns (**i**–**l**).

To ensure the fabricated wave shape matches with the mathematical simulation, the wavy pattern is measured and compared with the theoretical result. A contact profilometer is used to obtain the profile of the fabricated micro-wavy patterns. After considering the surface properties of the material, the stylus force in the profilometer was set to the minimum value of to 0.03 milligrams. Four types of wavy pattern – semi-sinusoidal wave, inverse semi-sinusoidal wave, triangular wave and trapezoidal wave - were selected for testing purposes. Figure 5(a–d) show curves in four groups; each group includes the simulated curve based on mathematical model (top), the measured curing shape (center), and a comparison plot (bottom). The grayscale exposure images are shown in Fig. 5(e–h). The digital microscope images of the corresponding wavy pattern are given in Fig. 5(i–l). All of the wavy features shown in Fig. 5 illustrate good agreement between the mathematical simulation and the actual surface measurements. Therefore, the established mathematical model and the corresponding grayscale exposure image can be used to accurately create a desired wavy pattern.

**Figure 6:**
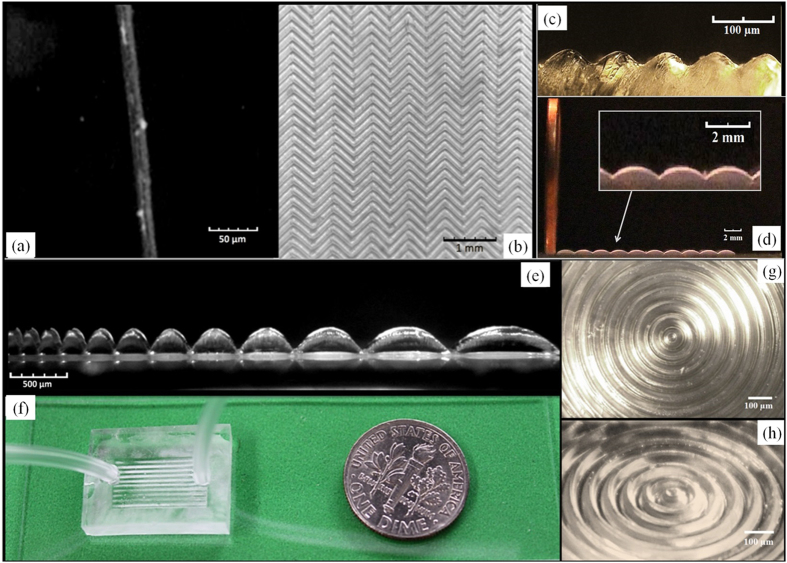
(**a**) The digital microscope image of a single wave. (**b**) The microscope image of the fabricated wavy-herringbone pattern. (**c**) The digital microscope image of the wavy pattern on PDMS after replication. (Side view) (**d**) Image of the wavy pattern with a wavelength of 2100 μm. (Comparing with a penny in thickness of 1.52 mm) (**e**) Microscope image of the wavy pattern with the increasing gradient of the wavelength. (Ratio of the gradient change is 120%) (**f**) A microfluidic device with wavy patterns. The digital microscope image of the fabricated wavy concentric circles patterns in (**g**) top view and (**h**) oblique view, respectively.

The current experimental setup is capable of generating wavy patterns with wavelengths ranging from 12 μm (Fig. 6(a)) to 2100 μm (Fig. 6(d)). Wavy patterns of even larger wavelength could be achieved by modifying the optic system to adjust the projected pixel size. The ratio of the amplitude to wavelength has an approximate upper limit of 300%, while most common method has an approximate 30% ratio limitation^8^. This method provides an easy way to fabricate three-dimensional wavy structures according to customizable 2D patterns, such as herringbone (Fig. 6(b)), and concentric circles (Fig. 6(g,h)). Wavy patterns with different wavelengths can be fabricated simultaneously, such as a wavy pattern with a gradient of wavelength (Fig. 6(e)). A customized exposure pattern for any profile can be created by simply making CAD drawings and selecting the target wave shape through numerical modeling. The UV curing process takes less than 20 seconds, which speeds up the fabrication process significantly compared to existing methods. The entire fabrication process -from a preliminary sketch to a ready-to-use micro-feature device can be accomplished in less than 30 minutes. Features generated using this method can be further embedded into microfluidic devices for more extensive applications. Our polymerized photocurable material is appropriate for FDTS-treatment, which facilitates the replication and separation of the PDMS molding (Fig. 6(c)). As such, a completely customized microfluidic device with wavy patterns (Fig. 6(f)) can be created within a few hours without access to clean room or commercial photolithography equipment using this method.

### Application: Cell Capture Test

To demonstrate a potential application for this micro-feature fabrication technique, microfluidic devices with pure wavy and wavy-herringbone patterns were made to capture circulating tumor cells (CTCs).

In the recent decade, microfluidic devices have been widely used for CTC detection, as summarized in several comprehensive review papers^21^,^22^. In our lab, a microfluidic device with integrated with wavy-herringbone patterns has been developed for CTC isolation with highly efficient and selectivity. With the advantage of flexible design and short processing time, the mask-free grayscale lithography method fits the need of a vast array of varying designs for CTC detection. In the section below, the application of a wavy-herringbone pattern printed by grayscale method in CTC detection has been described.

HCT-116 cells were selected as the target CTCs for the microfluidic test. Cells were cultured with McCoy’s 5a supplemented with 10% fetal bovine serum (FBS) and 1% Penicillin/Streptomycin. Incubation was obtained at 37 °C in 5% CO2 with media refreshed every 2–3 days. Before microfluidic tests, CTCs were detached from the flask through 5 minutes’ incubation in 0.05% Trysin-0.53 mM EDTA. Cells were then diluted in 4 mg/ml alginated PBS solution with a concentration around 10^5^/ml.

A standard protocol for coating anti-EpCAM was adopted to functionalize the microfluidic chip^23^. Before the cell flow test, the device was incubated with 5% BSA solution for 30 minutes and then flushed with PBS solution. Cell solution was then injected into the microfluidic chip under a certain flow rate through a syringe pump (Harvard Apparatus). After 5 minutes’ flow at a rate of 2 ml/hr, PBS solution was used to flush out all free cells at 2 ml/hr. Both regular microscope images and fluorescent images DAPI staining images were used to identify if all captured objects were CTCs.

**Figure 7:**
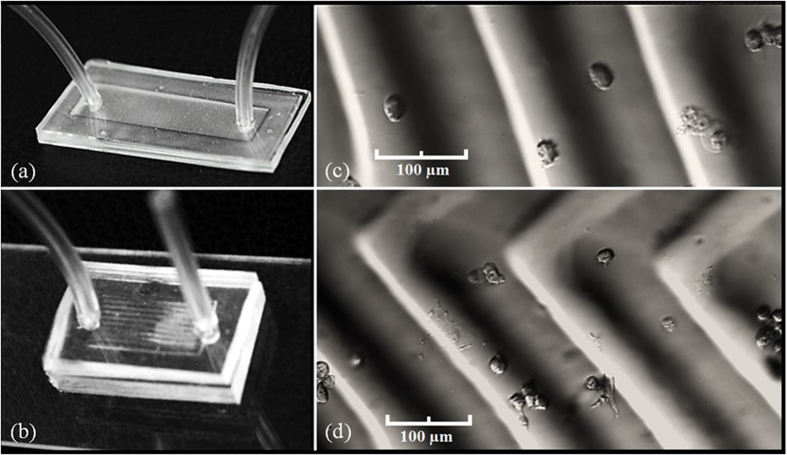
Microfluidic devices with (**a**) pure wavy pattern and (**b**) wavy-herringbone pattern, respectively. Microscope image of captured CTCs in microfluidic devices with (**c**) pure wavy pattern and (**d**) wavy-herringbone pattern, respectively.

Figure 7(a,b) illustrate the flow system. The printed device is the typical size of a microfluidic device. Distributions of CTCs after the flow test in our pure wavy and wavy-herringbone patterns are shown in Fig. 7(c,d), respectively. Due to the vortex induced in the microfluidic device, CTCs are captured both in the ridge and trough sections on the patterns.

Compared to previous methods in generating wavy patterns for CTC capture applications, this fabrication technique shows advantages of fast and precisely tuning the geometry parameters, as it is essential in determining the optimized settings of the CTC capture. In addition, the wavelength usually exceeds 100 μm in optimized CTC capture work^24^,^25^. The method presented in this paper is capable of fabricating wavy patterns with a wavelength greater than 100 μm, which cannot be achieved by some standard techniques^8^,^9^.

## Conclusion

This study provides a method of generating highly customizable, precisely controlled micro-wavy patterns. Compared with current approaches^8^,^9^,^10^, this method is capable of generating patterns with much wider range of wavelengths (12 μm to 2100 μm) and higher amplitude-to-wavelength ratio (up to 300%). Furthermore, since this method does not require a clean room or expensive lithography equipment, it brings not only high efficiency but great convenience and low cost to the fabrication of lab-on-a-chip devices with complex patterns. This grayscale lithography approach of generating surface patterns can also be developed as a versatile tool in surface studies. To achieve wavy-patterns with a wavelength shorter than 10 μm, more precise optical systems need to be introduced to reduce the optical interference of the light reflected from the DMD micro-mirrors.

## Experimental Methods

The first step in fabrication of micro-wavy patterns using this method is to adjust the top surface of the glass substrate to the focus level of the optical projecting system. Then transparent photocurable resin is poured inside the substrate container. During the pattern exposure process, the exposed part of the resin is cured and will adhere to the bottom glass substrate. After exposure, the glass substrate, containing the printed pattern and any uncured resin, is immersed into acetone to develop for 15 minutes. Isopropyl Alcohol 99% can also serve as the developer, but it takes a longer time to dissolve the uncured resin (30–45 minutes) and is unable to completely remove uncured resin if the wavelength of the features is particularly small. The substrate is then taken out of the acetone and dried off completely. A post-curing process uses a strong UV light source with a wavelength of 365 nm to further harden the polymerized patterns. The post-curing device in this experimental setup is an OmniCure s1500 that is capable of radiating 365 nm-wavelength UV light with high intensity irradiance of 23 W/cm^2^.

To make a microfluidic device by replicating the fabricated features with PDMS, the pattern surface is oxygen plasma treated and coated with a layer of 1H,1H,2H,2H-Perfluorodecyltrichlorosilane (FDTS) (Alfa Aesar) to make it hydrophobic. PDMS devices are then replicated from the FDTS-treated substrates and assembled into an integrated microfluidic chip. If the target pattern is the inverse shape of the exposure pattern, only one PDMS replication process is necessary. If the target pattern is the same as the exposure pattern, the final PDMS device needs to be formed from the PDMS substrate which has in turn been formed from the original device produced by the lithography process.

## Additional Information

**How to cite this article**: He, R. *et al.* Generation of Customizable Micro-wavy Pattern through Grayscale Direct Image Lithography. *Sci. Rep.* **6**, 21621; doi: 10.1038/srep21621 (2016).
